# The Evolution of Phenotypic Plasticity in Response to Temperature Stress

**DOI:** 10.1093/gbe/evaa206

**Published:** 2020-10-06

**Authors:** Francois Mallard, Viola Nolte, Christian Schlötterer

**Affiliations:** Institut für Populationsgenetik, Vetmeduni Vienna, Austria

**Keywords:** temperature adaptation, *Drosophila*, gene expression, phenotypic plasticity, experimental evolution

## Abstract

Phenotypic plasticity is the ability of a single genotype to produce different phenotypes in response to environmental variation. The importance of phenotypic plasticity in natural populations and its contribution to phenotypic evolution during rapid environmental change is widely debated. Here, we show that thermal plasticity of gene expression in natural populations is a key component of its adaptation: evolution to novel thermal environments increases ancestral plasticity rather than mean genetic expression. We determined the evolution of plasticity in gene expression by conducting laboratory natural selection on a *Drosophila simulans* population in hot and cold environments. After more than 60 generations in the hot environment, 325 genes evolved a change in plasticity relative to the natural ancestral population. Plasticity increased in 75% of these genes, which were strongly enriched for several well-defined functional categories (e.g., chitin metabolism, glycolysis, and oxidative phosphorylation). Furthermore, we show that plasticity in gene expression of populations exposed to different temperatures is rather similar across species. We conclude that most of the ancestral plasticity can evolve further in more extreme environments.

Significance

The role of phenotypic plasticity during adaptation to novel environments is actively discussed in the scientific community. We addressed the question of the evolution of plasticity during adaptation to a novel temperature regime using the powerful experimental *Drosophila* system in a controlled laboratory environment. Building on robust statistical analyses with replicated populations, which evolved for up to 64 generations, we demonstrate that phenotypic plasticity rapidly changes in evolving *Drosophila* populations. With evolution enhancing the plasticity of the ancestral natural population, we suggest that natural variation for gene expression plasticity has been driven by selection.

## Introduction

Phenotypic plasticity is of great interest in ecology and evolution, because it describes the ability of single genotypes to produce distinct phenotypes in different environments (Pigliucci 2001). When populations encounter environmental change, plastic traits will result in phenotypic alterations without genetic response (Price et al. 2003). Of particular importance are those adaptive plastic responses where the altered phenotype is associated with higher fitness, because they provide a selective advantage in variable environments (Charmantier et al. 2008; Nussey et al. 2005; Suzuki and Nijhout 2006; Ghalambor et al. 2007; Dey et al. 2016) or during adaptation to a rapid environmental shift. Phenotypic plasticity is well documented for a broad range of phenotypes including morphological or life-history traits (West-Eberhard 2003; Whitman and Ananthakrishna 2009). The technological advances in quantifying gene expression levels for entire transcriptomes have shifted the emphasis to gene expression patterns because many traits/phenotypes can be accurately quantified in a single experiment (Chen et al. 2015a; Zhao et al. 2015; Huang and Agrawal 2016).

Despite the conceptual appeal of adaptive plasticity in natural populations, our understanding of phenotypic plasticity in natural populations is still in its infancy (Pigliucci 2005; Merilä and Hendry 2014; Forsman 2015; Hendry 2016). In addition to adaptive plasticity, traits may be plastic in natural populations for other reasons: 1) neutral plasticity: variation in the trait has no fitness consequences (Via 1993) 2) deleterious plasticity: variation in the expression of the trait may be deleterious and selection operates to minimize it (Dewitt et al. 1998; Ghalambor et al. 2007). The comparison of populations in a common garden experiment is an intuitive and popular approach to infer the selective forces operating on plasticity (Merilä and Hendry 2014; Levis and Pfennig 2016). Nevertheless, the link between plasticity and adaptation is only correlative and may arise from other changes, not related to adaptation to the environmental contrasts.

Experiments relying on standing genetic variation to study the evolution of plasticity are well-placed in the framework of genetic accommodation (Braendle and Flatt 2006): complex traits with multiple contributing loci can respond quickly to environmental shifts. Hence, phenotypic plasticity could be rapidly modulated in response to selection. Exposing natural populations to more extreme environments provides clear predictions about the evolution of plasticity (Chevin and Hoffmann 2017). Although random changes in plasticity are expected under neutrality, in the case of deleterious (costly) plasticity, reduced plasticity is predicted (counter-gradient evolution). An increase in plasticity is expected when plasticity is adaptive: genetic changes in the novel environment will reinforce the ancestral plasticity (Ghalambor et al. 2007; Ho and Zhang 2018). No change in plasticity is difficult to interpret because it may reflect absence of genetic variation, but also weak selection or neutral plasticity result in the same outcome. Experimental evolution is a powerful approach to distinguish between random and directed changes in plasticity because environmental conditions can be tightly controlled and replicated experiments provide more reliable results.

In *Drosophila*, the evolution of gene expression plasticity has been studied for a range of different environmental stressors, ranging from alcohol to heavy metals and temperature (Levine et al. 2011; Yampolsky et al. 2012; Zhou et al. 2012; Chen et al. 2015a; Zhao et al. 2015; Clemson et al. 2016; Huang and Agrawal 2016; Porcelli et al. 2016). Natural *Drosophila* populations are exposed to daily and seasonal temperature fluctuations (Bergland et al. 2014; Machado et al. 2016), making this a particularly relevant abiotic factor in the context of phenotypic plasticity (Angilletta and Angilletta 2009). Measuring gene expression of a single heterozygous *Drosophila melanogaster* genotype at four different temperatures showed that 83% of the expressed genes exhibit a plastic expression pattern when exposed to a temperature gradient ranging from 13 °C to 29 °C (Chen et al. 2015b). The variation in gene expression plasticity of natural *Drosophila* populations along latitudinal clines (Zhao et al. 2015; Porcelli et al. 2016) suggests that some of the plastic responses are driven by selection.

**Fig. 1.**
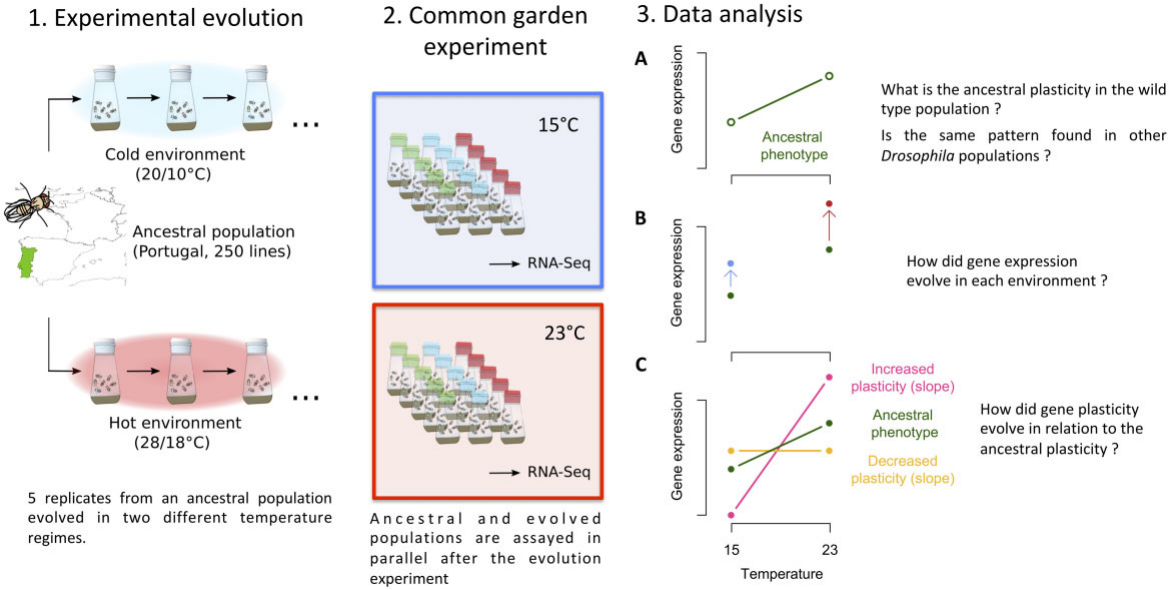
Experimental design. (*A*) We evolved two sets of five populations in either cold or hot laboratory environments for 39 and 64 generations, respectively. (*B*) We measured gene expression in two common gardens, where the evolved populations together with the ancestral one were phenotyped at either 15 °C or 23 °C. (*C*) Gene expression analysis was done in three successive steps. (*a*) We first explored the plasticity of our ancestral population and compare it to existing data sets. (*b*) We investigated gene expression changes at 15 °C and 23 °C in the evolved populations. (*c*) We determined the evolved plasticity by measuring each of the evolved populations in both temperature regimes. The evolved plasticity is compared with the ancestral one.

We study the evolution of plasticity to infer the influence of high and low-temperature regimes on the plasticity of gene expression in *Drosophila simulans* using laboratory natural selection (Fuller et al. 2005, see experimental design in fig. 1). Specifically, we address the question how adaptation to more extreme temperatures modulates the plastic response of traits, which were already plastic in the founder population. We show that phenotypic plasticity does not prevent evolution. Rather, adaptation to more extreme temperature regimes increases the plastic response. In combination with clinal variation of gene expression in natural populations of both *D. simulans* and *D. melanogaster* (Zhao et al. 2015), our data provide convincing experimental evidence for adaptive phenotypic plasticity in a natural population.

## Materials and Methods

### Laboratory Natural Selection Procedure

The laboratory natural selection setup is detailed in Mallard et al. (2018). In brief, ten replicated *D. simulans* populations were setup from 250 isofemale lines collected in Northern Portugal in 2008. The replicated populations are maintained under two fluctuating temperature regimes (five replicates in each): either a hot (mean temperature 23 °C) or a cold treatment (mean temperature 15 °C). In each environment, the temperature changed with a 10 °C amplitude centered on the mean temperature synchronized on a 12/12 h light/dark cycle. The same maintenance regime was used for populations in both temperature environments, only adjusting for the increased developmental time in the cold environment. Every generation, 1000 flies are sampled from the eclosed flies and distributed over five fresh bottles containing 70 ml standard *Drosophila* medium. After two egg layings for 48 and 72 h in the hot and cold environment, respectively, adults were frozen. We preferentially used the second egg collection for the next generation to avoid selection for early fecundity. We previously showed that the selection regime results in higher fitness of the evolved populations (Mallard et al. 2018).

### Common Garden Experiment

Two parallel common gardens with identical experimental procedures were performed in a hot (23 °C) and a cold (15 °C) environment using eggs from the evolved populations at generation 39 (cold) and 64 (hot). Additionally, five replicates of the ancestral population were reconstituted from the founder isofemale lines. After two generations in the assayed environment, the second one with controlled larval density (300 eggs), we collected adults and separated the two sexes under shallow CO_2_. Flies were frozen in liquid nitrogen after a 24–36 h recovery period at 2 pm (approximately 6 h after the start of the light cycle). During experimental evolution, the ancestral population was maintained at 18 °C in the form of isofemale lines. The small population size in the isofemale lines prevents adaptation to the culturing conditions and therefore, the reconstituted population reflects the ancestral population (Nouhaud et al. 2016).

### Gene Expression Analysis

For all 15 populations from both common garden temperatures, we generated two RNA-Seq libraries, each from different sets of 25–30 males. We extracted total RNA-Seq using the Qiagen RNeasy Universal Plus Mini protocol (Qiagen, Hilden, Germany) with DNase I treatment according to the manufacturer’s instructions. Quality control of the RNA was performed on agarose gels and the Qubit RNA HS or BR Assay kit (Invitrogen, Carlsbad, CA) for quantification. Strand-specific barcoded mRNA libraries were generated using the NEBNext Ultra Directional RNA Library Prep Kit for Illumina with a protocol modified to allow for a larger insert size than the default 200 bp. We purified polyA-mRNA from 3 μg total RNA and fragmented for 8 min. The 42 °C incubation step in the first-strand synthesis and the 16 °C step in the second-strand synthesis were extended to 30 and 90 min, respectively. Size selection for a target insert size of 330 bp was performed using AMPure XP beads (Beckman Coulter, Carlsbad, CA). PCR amplification followed the recommended protocol (NEB) with 12 PCR cycles and a 50 s. extension step. The final libraries were bead-purified, quantified with the Qubit DNA HS Assay kit (Invitrogen, Carlsbad, CA), and pooled in equimolar amounts. To reduce batch effects, we combined libraries from ancestral, cold-, and hot-evolved replicates and sequenced them in the same lane. Libraries were sequenced using a single-read 50-bp protocol on a HiSeq2500.

We trimmed the raw reads (quality threshold 20, minimum read length 40) using PoPoolation (Kofler et al. 2011). The trimmed reads were aligned to the *D. simulans* reference genome (Palmieri et al. 2015) with GSNAP (Wu and Nacu 2010) using a hadoop cluster. All subsequent analyses were performed in R (R Core Team 2019) including read counts (Liao et al. 2013) and differential gene expression (Robinson et al. 2010). We normalized gene expression levels with the TMM method, restricting our analysis to the genes with an overall mean expression above one count per million (CPM, 11,200 genes). We used negative binomial Generalized Linear Models (GLM) to estimate the effect of selection regime, temperature, and their interaction on gene expression. We then computed ad hoc contrasts to find differentially expressed genes between groups of interest using likelihood ratio tests (*glmLRT* in edgeR). This allows us to determine for each gene whether the difference in expression either between two groups of samples (such as the effect of temperature on a given evolved population) or for a linear combination of these groups (such as the difference between the reaction norms of two populations) is statistically significant. The Benjamini–Hochberg procedure was applied to control for false discovery rate (Benjamini and Hochberg 1995). All plasticity estimates as well as evolved differences between ancestral and evolved populations plotted in the manuscript are model fit values obtained from these contrasts.

When comparing the gene expression of evolved populations against the ancestral ones at a given temperature, we always used FDR <0.05 (unless specified differently). We allowed a higher rate of false positive when testing for reaction norms between ancestral and evolved populations (FDR <0.1). This was done because we restricted our analysis to genes that were already differentially expressed in at least one temperature with a stringent FDR. Once identified the genes showing a significant evolution of their reaction norms, we compared the absolute value of the ancestral and the evolved reaction norms to distinguish between cases of reduced and increased plasticity. Gene ontology enrichment was performed with Gorilla (Eden et al. 2009) using the complete list of retained genes (*n* = 11,200) as background data set and an FDR <0.05. We compared the number of genes that evolved increased or decreased plasticity in the hot-evolved populations using a generalized linear model with a binomial distribution. The estimated probability was compared with the 0.5 using a Wald test.

In a second GLM, we analyzed the replicate specific evolutionary response. We considered only the samples from the ancestral and the hot-evolved populations and each evolved population was treated as a different level of the “selection regime” factor. The model formula was similar to the previous one but this latter factor contained six levels (Ancestral and each of the five hot-evolved replicates). We processed as described above to detect genes with evolved differential expression.

### Detection of False-Positive Genes with Increasing Plasticity

To avoid false positives, we restricted our set of candidate genes to those with a significant change in expression in at least one of the two environments (15 °C or 23 °C) and a significant interaction effect. The rationale can be explained by considering genes that evolved in expression in only one environment, but remained unchanged in the second environment. Adding some minor random noise could either result in a positive or negative correlation of the expression changes in both temperatures. Because negative correlation increases the significance in the interaction test, it may be possible that such random fluctuations could bias our results toward the observed excess of genes with increased plasticity. To rule out that such a potential bias affected our results, we performed an additional test contrasting the ancestral plasticity and the plasticity of a hypothetic population that would have evolved its expression only at one temperature (i.e., replacing the expression levels of the evolved population in the second environment by the ancestral values). For all genes with a significant change in plasticity, we also detected a significant change in plasticity when we considered only the expression change in only one environment. We conclude that none of these genes are false detected due to a random measurement error in the second environment.

### RNA-Seq Quality Control

We performed several analyses to test the quality of each library. We first estimated heterogeneity in coverage (3ʹ bias) of the 20% longest genes of the *D. simulans* annotation using the geneBody coverage tool implemented in the RSeQC package (Wang et al. 2012). Following Mallard et al. (2018), we removed strongly biased libraries (12 libraries in total). Additionally, we quantified the expression of 12 chorion and yolk protein genes to identify female contamination due to sexing mistakes or sample swap. We excluded libraries showing a total log_2_ normalized expression of these genes higher than eight (four libraries, see supplementary fig. S6, Supplementary Material online). These four libraries contained at least 16 times the number of transcripts in the remaining libraries (see supplementary fig. S6, Supplementary Material online). After removing the biased and contaminated libraries, a total of 44 libraries remained for the analysis (less than 1.5 samples per population). Out of these 44 libraries, 16 combinations of populations and treatment had only one library left and 14 had 2 libraries. We retained only one measurement per population (*n* = 30) by summing the gene counts of samples coming from the same population. Before pooling the libraries, we visually inspected the samples using multidimensional scaling plots (supplementary fig. S1, Supplementary Material online). These plots inform about pairwise distance between samples. Although the replicates within a temperature regime were not well separated, robust differences between the ancestral and the two groups of evolved populations were seen. The number of mapped reads for each sample can be found in supplementary table S6, Supplementary Material online.

## Results

We measured the gene expression patterns of our ancestral population and the two evolved populations in two parallel common gardens at 15 °C and 23 °C. The analysis of evolution of gene expression plasticity is complex and we followed a three-step analysis as described in figure 1.

### Gene Expression Plasticity in the Ancestral Population

We determined temperature-mediated plasticity of gene expression by exposing the ancestral, hot-evolved, and cold-evolved population to 15 °C and 23 °C (fig. 1.3*A*). As expected from previous studies (Zhou et al. 2012; Chen et al. 2015b), the expression of a large number of genes was modulated by temperature.

Downregulated genes, which are expressed at lower levels at 23 °C than at 15 °C in the ancestral population, are enriched for several GO categories including chitin-based cuticle and transmembrane transport genes (supplementary table S1, Supplementary Material online). Eighty-nine (83%) of the significant GO terms are also identified among the genes decreasing in expression at higher temperatures in *D. melanogaster* (out of 107 GO terms classified in Chen et al. [2015b]). This overlap is probably conservative, because the sex of the flies analyzed and the temperature regimes differed between studies (Chen et al. [2015] measured females in four different temperatures). Interestingly, Zhao et al. (2015) found that chitin genes were among the top plastic genes shared between *D. melanogaster* and *D. simulans.* In particular, the category “structural constituent of chitin-based cuticle” was consistently identified for genes decreasing with temperature across all three studies.

Genes that are more highly expressed at 23 °C than at 15 °C in the ancestral population (upregulated genes) are enriched for genes involved in translation, including a large number of ribosomal genes. Out of 21 GO terms, which were also enriched in Chen et al. (2015), 18 are classified as increasing in both analysis (supplementary table S2, Supplementary Material online). None of these categories were reported in Zhao et al. (2015).

Such highly consistent gene expression changes across different experiments suggest a highly robust pattern of plasticity, which is conserved not only among populations, but also between species.

### Evolution of Gene Expression in the Focal Temperature Regime

Only a small number of genes were differentially expressed in populations evolved in the cold environment when compared with the ancestral population (see supplementary table S3, Supplementary Material online FDR < 0.05; 42 genes at 15 °C) (fig. 1*.3B*). A quite different pattern was observed for the hot-evolved populations. In the comparison to the ancestral population, 725 genes (see supplementary table S4, Supplementary Material online) were differentially expressed at 23 °C. The small impact of adaptation to cold temperature may be the consequence of fewer generations in the new environment compared with the hot-evolved populations. But we cannot distinguish this effect from temperature-specific effects triggering a more pronounced evolution in the hot environment.

### Evolution of Gene Expression Plasticity

With about 32% (*n* = 3,602, FDR <0.05) of the expressed genes being differentially expressed between the two assaying temperatures, the cold-evolved population was slightly less plastic than the ancestral population (*n* = 4,352, 39%, see supplementary table S1, Supplementary Material online) (fig. 1*.3C*). The hot-evolved population had about 44% (*n* = 4,909) plastic genes, which corresponds to about 15% more differentially expressed genes than the other two populations. These differences remain stable even when controlling for the overall library sizes by downsampling (see supplementary fig. S3, Supplementary Material online).

**Fig. 2.**
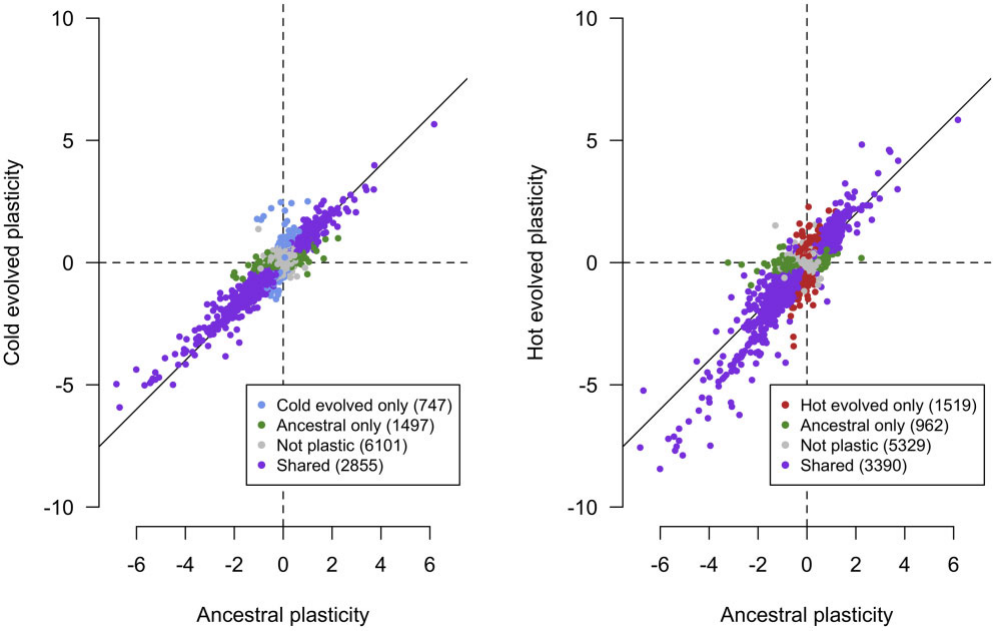
Evolution of gene expression plasticity after selection in cold (left) or hot environments (right). Plasticity is measured as the log_2_ fold change of gene expression at 15 °C and 23 °C. We compare the plasticity of the ancestral population (*x* axis) plotted against the plasticity of the evolved populations. Overall, the pattern of gene expression plasticity is conserved for many genes (purple). Genes that are significantly plastic in only one population have lower log_2_ fold changes in other population (green, blue, and red dots). Despite this overall conservation of plasticity, highly plastic genes tend to deviate from the solid line in the hot-evolved populations (slope = 1) indicating an increased plasticity.

We evaluated the evolution of plasticity by correlating gene expression plasticity (log_2_FC between 15° and 23 °C, that is the slopes shown in fig. 1, panel *3C*) in the ancestral population with the plasticity in the evolved populations. If the plasticity did not change during evolution, a high correlation is expected. Indeed, the plasticity was highly correlated between ancestral and evolved populations (Pearson correlation coefficients: 0.91 [cold evolved] and 0.89 [hot evolved], fig. 2). Despite this overall conservation of gene expression plasticity, a closer inspection of figure 2 (right panel) shows that for some genes plasticity changed after evolution in the hot environment, but the direction of plasticity is not affected (i.e., the plasticity became more extreme).

In the cold-evolved replicate populations, only a small subset of the genes that evolved a change in expression at 15 °C or 23 °C displayed a significant difference in the plasticity relative to the ancestral population (two at 15 °C and two genes at 23 °C, FDR <0.1).

Among the genes that evolved gene expression differences in the hot-evolved populations either at 15 °C or at 23 °C (*n* = 930), we distinguished three different classes: 1) genes with significant change in plasticity (325 genes, FDR <0.1); 2) genes with small differences in the magnitude of gene expression differences (log_2_FC) between the evolved and the ancestral population in each environment—here, a reliable detection of changes in plasticity or constitutive expression differences is not possible; 3) genes with no change in plasticity, but constitutive expression differences (i.e., a change in the same direction at both temperatures, FDR < 0.05, *n* = 50). This third class of genes was enriched for oxido-reduction processes suggesting a global downregulation of detoxification genes (FDR < 0.1, 6 cytochrome p450 genes, 2 UDP-glucuronosyltransferases). Because some of these genes were also downregulated in the cold-evolved populations (23 genes using FDR <0.1 including 6 p450 genes, see supplementary table S3, Supplementary Material online), we conclude that their constitutive change in expression is not directly related to absolute temperature but a response to either temperature stress or to adaptation to shared environmental conditions.

Among the 325 genes with a significant evolution of plasticity, we noticed significantly more genes with increased plasticity (*n* = 241) than with decreased plasticity (*n* = 84, *P* < 0.001). This result is not biased by ancestrally nonplastic genes that cannot decrease plasticity: the ratio of genes with increased plasticity to genes with decreased plasticity does not change when only ancestrally plastic genes are analyzed (log_2_FC >1 in the ancestral population, *n* = 62 and 20, respectively, *P* < 0.001). No GO categories were enriched for genes with reduced phenotypic plasticity. In contrast, genes with increased phenotypic plasticity were enriched for several GO terms (116 processes, 34 functions, and 28 components). Because the different number of genes in both categories may have affected the enrichment tests, we randomly selected multiple sets of 85 genes among the 242 significant ones and performed the GO analysis for each set. We obtained significantly more enriched processes genes that evolved an increased plasticity (20 bootstrap iterations, *P* < 0.0004; mean number of enriched processes 18.3). Two particularly prominent classes of GO terms were either related to cuticle formation and chitin production or metabolism including the electron transport chain and glucose metabolic processes.

**Fig. 3.**
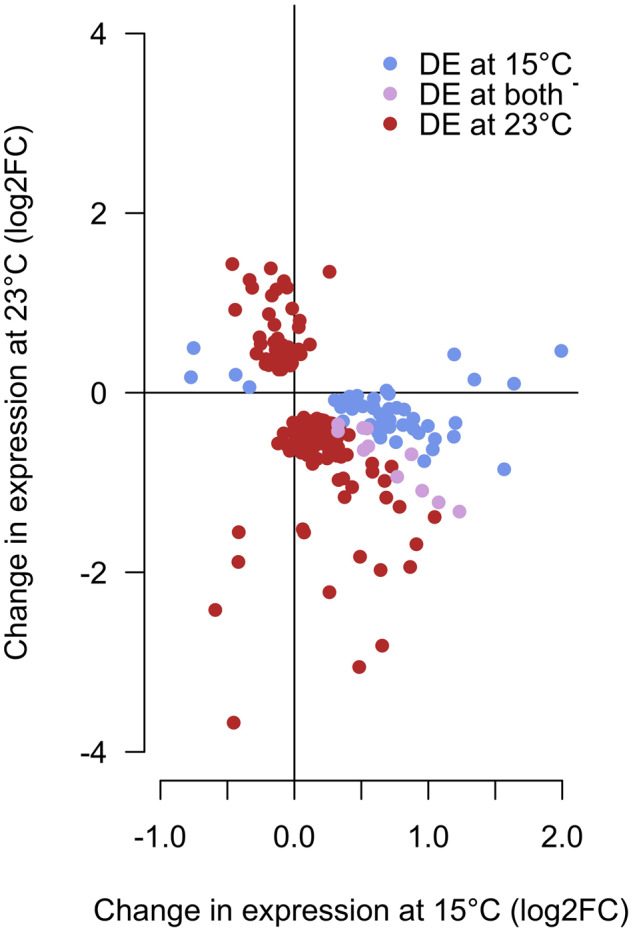
Log_2_FC in gene expression of genes increasing in plasticity during evolution in the hot environment and being differentially expressed in at least one assayed temperature. The *x* and *y* axes show the impact of adaptation to a hot environment on the gene expression at 15 °C and 23 °C relative to the ancestral population. Most of these genes evolved a change in expression in the opposite direction (*P* < 0.0001) and are therefore located in the top-left and bottom-right quarters of the plot.

**Fig. 4.**
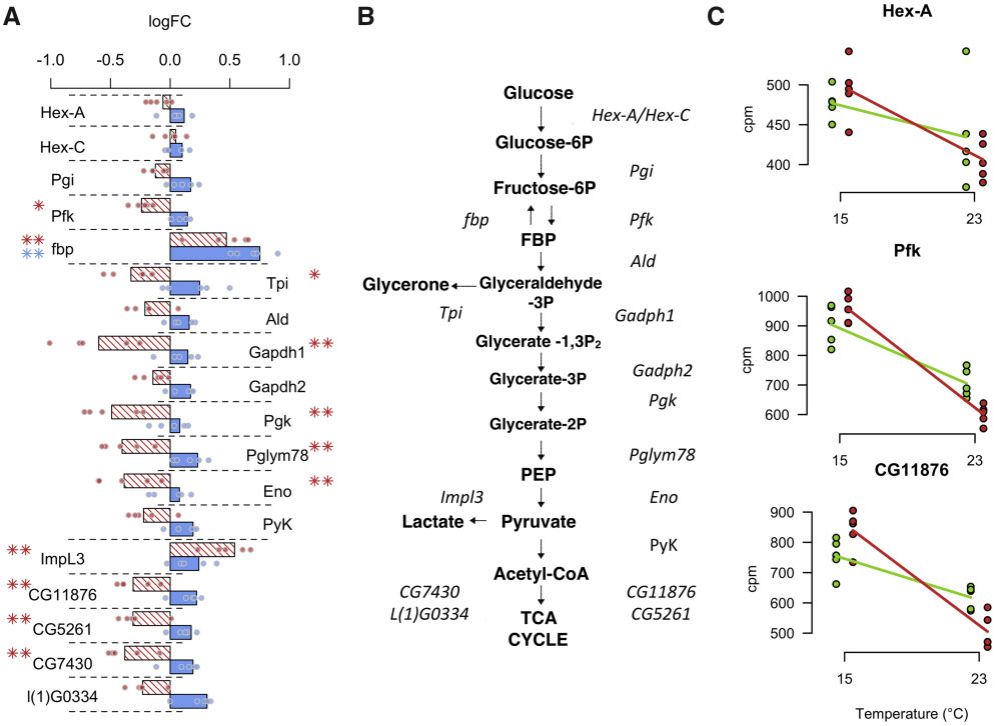
Evolution of plasticity in the glycolysis pathway. (*A*) Bar chart of the log_2_FC of gene expression evolution from figure 2. The difference between the ancestral and the hot-evolved populations measured at 15 °C (blue) and 23 °C (red) are shown. Single dots superimposed on the bar show the divergence of five hot-evolved replicates from the mean ancestral expression. (*B*) Glycolysis pathway with the main regulatory enzymes from *A*. Most genes involved in glycolysis are significantly downregulated at 23 °C (**FDR <0.05, *FDR <0.1). Even for comparisons with no statistically significant difference, most of the genes downregulated at 23 °C are upregulated at 15 °C. (*C*) Expression plasticity is highly reproducible across replicates. Three enzymes of the glycolysis pathway illustrate the highly consistent response across all five replicates. The ancestral replicate populations are indicated by green dots and the hot-evolved populations by red dots (all genes are shown in a supplementary file, Supplementary Material online). Lines indicate plasticity based on the mean expressions values of the five replicates.

For most of the genes that evolved a difference in gene expression between ancestral and evolved populations, there is a significant change at only one temperature (FDR <0.05). Nevertheless, we noted a strong negative correlation for the sign of the expression differences between hot evolved and founder populations at 15 °C and 23 °C (fig. 3, χ^2^_1,241_ = 133, *P* < 0.0001, see also supplementary fig. S4, Supplementary Material online for a complementary test), suggesting that evolution modulated the temperature sensitivity of gene expression. This negative correlation is particularly pronounced for genes involved in energy production (see fig. 4 and supplementary fig. S2, Supplementary Material online for glycolysis and oxidative phosphorylation) but also for chitin-related genes.

On the other hand, genes that evolved a decreased plasticity show a much weaker correlation of the sign of expression change between temperatures (χ^2^_1,84_ = 2.8, *P* = 0.09).

### Replicate Specific Evolution

Our previous analysis is looking for significant changes in expression across five independently evolved populations. Yet, it does not inform us about the parallel evolution of each population. We addressed this by analyzing each replicate independently to detect genes evolving increased or decreased phenotypic plasticity.

In each evolved population, we detected genes that evolved plasticity (range: 85–249, mean =134, 409 genes in total). Only 18 genes were significant in all 5 replicates and 272 in only a single population. Similarly to what we found in the main analysis, more genes displayed increased plasticity (ranging 63–175, *n* = 318) than decreased plasticity (ranging 26–41, *n* = 101). This observation is very consistent across populations: a gene that evolved plasticity in one replicate is found significant in another one with the same frequency (41% and 45% for genes decreasing and increasing plasticity, respectively). For changes in reaction norm, the consistency across replicates is highly dependent on the direction of change. We observed a low correlation for genes that evolved decreased plasticity (mean *r*^2^ = 0.03, see supplementary fig. S5, Supplementary Material online) whereas genes that increased plasticity were highly correlated among replicates (mean *r*^2^ = 0.77, see supplementary Material online fig. S5, Supplementary Material online).

**Fig. 5.**
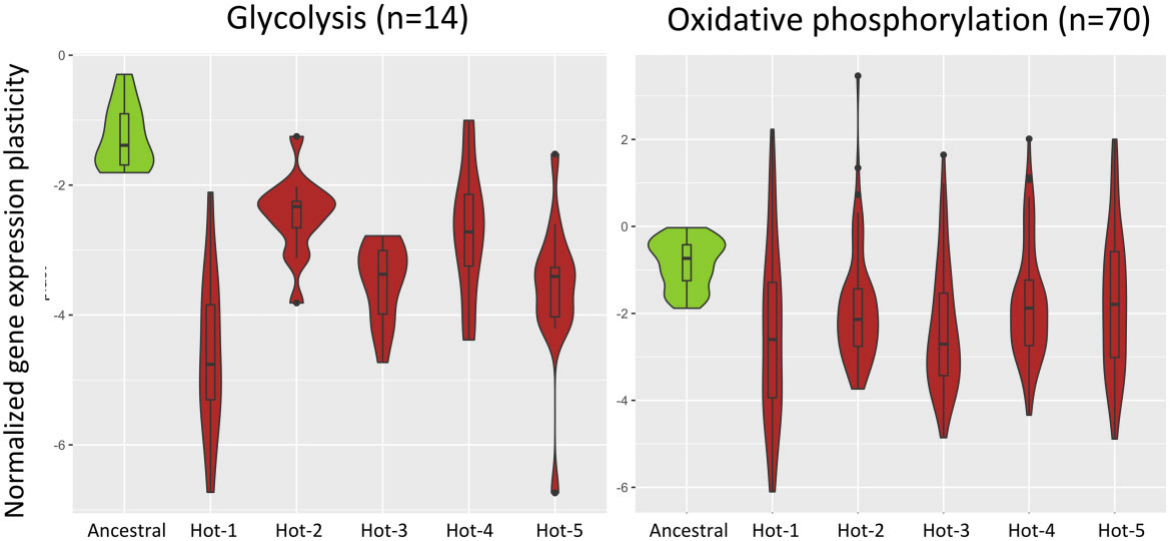
Highly consistent downregulation of glycolysis and oxidative phosphorylation plasticity across all five hot-evolved replicates. The difference in expression between 15 °C and 23 °C of the ancestral population (green) is higher than in each of the evolved populations (red) indicating more negative reaction norms. We only show genes from the glycolysis and oxidative pathways that were ancestrally downregulated (right panel, *n* = 14, left panel, *n* = 70).

A GO enrichment analysis at the replicate level showed that only chitin-related gene ontologies were significantly enriched in all five evolved populations (see supplementary table S5, Supplementary Material online). The increased plasticity of the metabolism-related genes was only significantly overrepresented in the first replicate. Nevertheless, we attribute this mainly to a lack of statistical power: the increase in plasticity for the genes involved in glycolysis and oxidative phosphorylation is consistent across all replicates (fig. 5).

## Discussion

### Only Limited Counter-Gradient Evolution

Only very few studies were able to address the evolution of gene expression over short evolutionary time scales. The adaptation of *D. melanogaster* to salt and cadmium-enriched medium (Huang and Agrawal 2016) showed that gene expression plasticity evolved, but in the opposite direction to the plasticity seen in the ancestral populations. These food supplements are novel environmental conditions, which are very rarely encountered by fruit flies in their natural environments. The authors proposed that this counter-gradient evolution could be explained by the selection on phenotypes that are only beneficial under these extreme conditions, but not in the environments typically encountered by *Drosophila*: the plastic response would correspond to a “stress” that is no longer expressed when population are adapted to this new environment. This is in sharp contrast to the experimental design of this study. Temperature is one of the most important environmental factors driving local adaptation in ectotherms (Angilletta and Angilletta 2009; Fuller and al. 2005). This applies also to *Drosophila* (Klepsatel et al. 2013; Bergland et al. 2014; Zhao et al. 2015; Machado et al. 2016) where significant clinal variation is seen on the genomic and transcriptomic level (Hoffmann and Weeks 2007; Zhao et al. 2015; Machado et al. 2016). Only a moderate fraction of genes (25% of all genes with evolved plasticity) that experienced counter-gradient evolution, that is, a decrease in the slope of the reaction norm. Interestingly, these genes were not enriched for functional categories and did not evolve consistently across our replicates. Thus, we failed to find biological processes for which gene expression plasticity would be strongly maladaptive. More likely, the gene expression of these genes is not well-adapted, possibly due to pleiotropic functional requirements, which are relaxed in the laboratory environment. The large fraction of genes for which the ancestral plasticity evolved to more extreme values suggests that the laboratory conditions match many ecologically relevant forces encountered by natural *Drosophila* populations.

In our study, we contrasted whole organisms gene expression across environments—a common practice in the study of gene expression evolution in *Drosophila* (Levine et al. 2011; Yampolsky et al. 2012; Zhou et al. 2012; Chen et al. 2015a, 2015b; Zhao et al. 2015; Clemson et al. 2016; Huang and Agrawal 2016; Porcelli et al. 2016). Nevertheless, a potential problem is that during evolution allometric changes may occur—this is that the relative abundance of some cell types changes (Montgomery and Mank 2016). In fact, a recent study showed that females adapting to a new temperature regime also evolved allometric changes, whereas males were much less affected (Hsu et al. 2020). Although such allometric changes could affect gene expression means, the impact on plasticity is not clear. If the evolved allometric changes do not change with assaying temperature, no influence on the analysis of phenotypic plasticity is expected. On the other hand, if allometric changes are modulated by assaying temperature, this could be considered as an extended evolved phenotypic plasticity and will not affect our conclusions.

### Plasticity in Gene Expression Suggests Adaptive Plasticity

The evolutionary implication of phenotypic plasticity is a controversial topic with two extreme perspectives. With the same genotype expressing different phenotypes in response to the environment, it is often assumed that these phenotypes provide a higher fitness to their carriers (Via et al. 1995). If phenotypic plasticity results in a good match of phenotype and environment, this could even make genetic adaptation expendable (Charmantier et al. 2008). On the other hand, phenotypic plasticity of many traits may not contribute to fitness and reflects pleiotropic responses to environmental changes. This uncertainty about the evolutionary consequences has not yet been settled because of the difficulty to link plasticity with fitness advantage. Our study links the evolutionary response in a laboratory natural selection experiment to plasticity in the founder population. Out of 3,605 genes with plastic gene expression pattern after exposure to two temperatures, 327 genes (9%) changed plasticity after 59 generations. Reasoning that the hot laboratory environment is more extreme than the habitat of the founder population, genes with adaptive plasticity for temperature should evolve toward increased plasticity (Garland and Kelly 2006; Lande 2009). Consistent, with this expectation, 75% of the genes with evolved plasticity increased their environmental sensitivity. Genes with increased thermal sensitivity showed functional enrichment and were more consistent in their change across replicates. Our gene expression results are in line with the prevalence of genetic variation for thermal plasticity in natural *Drosophila* populations (Levine et al. 2011; Zhao et al. 2015, but see Clemson et al. 2016). The parallel evolution in plasticity suggests a selective advantage of populations with evolved plasticity, but our experiment cannot decide whether the evolved plasticity is providing the fitness advantage or it is a pleiotropic effect caused by the true target of selection. Finally, most of the genes with increased plasticity showed an opposite evolutionary response at 15 °C and 23 °C leading to the reinforcement of the ancestral plasticity which is expected in the case of adaptive plasticity (Ghalambor et al. 2007; Ho and Zhang 2018).

Future experiments, measuring individual flies gene expression would allow us to study the evolution of the trait gene expression plasticity. Comparing the trait distribution in the ancestral and evolved populations after the new trait optimum has been reached will provide further insights in the underlying adaptive architecture.

### Evolution of Plasticity Is More Frequent Than Constitutive Expression Changes

In hot-evolved populations, only 7% (52 out of 729) of the genes, which evolved a significant response relative to the founder population at 23 °C, showed a constitutive expression difference rather than an evolutionary change of plasticity. It is not clear if this predominance of plastic response reflects the design of the laboratory natural selection experiment, which involved daily temperature fluctuation or a correlated response to directional selection (Garland and Kelly 2006).

Although the flies evolved in a novel temperature regime with daily fluctuations, we measured gene expression in constant temperature regimes to avoid confounding effect of development at different temperatures. Classic examples for the persisting effects of short-term exposure to high temperatures are phenocopies. Short (<5 h) sensitive periods of *Drosophila* pupae result in different phenotypes depending on the developmental stage during exposure (Mitchell and Lipps 1978). Hence, even small differences in developmental timing could result in large phenotypic variation within or between populations. Thus, we opted for a constant temperature common garden. This strategy assured phenotypic measurements insensitive to daily temperature fluctuations, reflecting fixed temperature effects that are comparable to existing phenotype data. Given that the expression of most genes changes monotonically with temperature (Chen et al. 2015b), we anticipated that observed differences in reaction norm at 15 °C and 23 °C can be extrapolated to more extreme temperatures, such as 10 °C and 28 °C.

Although it is possible that the observed gene expression changes are not the direct target of selection, it would not challenge our claim that ancestral plasticity is likely to be adaptive: even if the evolution of the gene expression in our experiment is only correlated with the selected trait(s), the ancestral plasticity we observed at the gene expression level remains an indicator of adaptive plasticity because the direction of change is, by definition, the same between these correlated traits. The validity of our conclusions would only be challenged if during evolution these phenotypic correlations across temperature were broken. We consider this, however, unlikely as the temperature response is conserved across populations and various *Drosophila* species (see also Zhao et al. 2015).

We previously identified *SNF4Aγ* and *Sestrin* as targets of selection in the same hot-evolved populations (Mallard et al. 2018). Both genes are associated with activity of AMPK, a key enzyme in metabolism regulation. Interestingly, the role of AMPK in thermal plasticity has been highlighted in marine invertebrates such as mussels and rock crabs that are regularly subjected to temperature variation during tides (Frederich et al. 2009; Jost et al. 2015). Moreover, mussels experience seasonal variation in thermal plasticity of AMPK activity (Jost et al. 2014), which is comparable to the evolution of plasticity in our evolved populations. In addition to metabolism, chitin synthesis was found to be plastic, which is shared with *D. melanogaster* (Chen et al. 2015b) and in the North American cline (both *D. melanogaster* and *D. simulans* [Zhao et al. 2015]). Chitin is involved in exoskeleton morphogenesis and its decreased synthesis may be associated with the temperature-induced size reduction in *Drosophila*. However, we did not find any evolution of body size during our experiment (data not shown). Alternatively, chitin is also essential for trachea formation (Moussian et al. 2005), and the evolution of its synthesis in our experiment could be linked with the decrease in metabolism gene expression.

Previous experimental evolution studies in *Drosophila* have found inconsistent results regarding the evolution of gene expression plasticity (Yampolsky et al. 2012; Huang and Agrawal 2016) and it is not clear if this inconsistency can be explained by different environmental stressors. On the other hand, it has been proposed that plasticity increases during the initial phase of adaptation to novel environments, followed by genetic assimilation (Lande 2009). In this theoretical scenario, also called “plasticity first” (Levis and Pfennig 2016; Levis et al. 2018), the genomic variation which encodes phenotypic plasticity is favored as a rapid phenotypic response. As a consequence, selection signatures are expected for genes modulating plasticity, rather than in cis-regulatory variation of genes with modified gene expression patterns.

In the context of the current ongoing climate change, the role of phenotypic plasticity has been widely discussed—does plasticity favor or limit genetic adaptation (Merilä and Hendry 2014; Sgrò et al. 2016; Vázquez et al. 2017; DeBiasse et al. 2018)? As recently stated by Kelly (2019), if plasticity is a major contributor of adaptation to climate change, then the amount of available genetic variation for plasticity could be a reliable predictor of a population vulnerability. In particular, for *Drosophila*, the potential of plasticity for attenuating the impact of climate change has been challenged. Thermal plasticity does not correlate with latitude (Sørensen et al. 2016) and did not respond to laboratory natural selection in higher-order phenotypes when submitted to stable or fluctuating environments (Manenti et al. 2015; Fragata et al. 2016). Our experiments provide some important insights into this debate. The highly parallel response in replicated populations demonstrates that genetic variation in thermal plasticity is a reservoir for adaptation in novel thermal environments.

Because we studied plasticity after only a moderate number of generations, our study is not informative for more long-term evolutionary processes. Recently, it has been shown that on the long term, this evolutionary response could lead to extinction unless a small number of genetic loci are involved (Nunney 2016). Whether a phase of genetic assimilation will follow this initial increase in plasticity will depend on the availability of the relevant variation. If such variants are still segregating, it could be informative to test our experimental populations at later generations. If new mutations are required, experimental evolution in *Drosophila* may not be well-suited to address this question because the spread of new mutations is rare (Burke et al. 2010).

## Supplementary Material

Supplementary data are available at *Genome Biology and Evolution* online.

## Supplementary Material

evaa206_Supplementary_Data
