# Supplementary material for: The Evolution of Phenotypic Plasticity in Response to Temperature Stress
Source: Genome Biol Evol. 2020 Oct 6;12(12):2429–40. doi: 10.1093/gbe/evaa206 (PMC7846148; doi:10.1093/gbe/evaa206)
Supplement: evaa206_Supplementary_Data [file evaa206_supplementary_data.zip › Mallard_Supporting_Information_Legends.docx]

**Supporting Information Legends**

Fig. S1: Multi-dimensional scaling plots of the different samples. Ancestral, Cold evolved and Hot evolved population are respectively labeled A[1-5], C[1-5] and H[1-5] and colored in green, blue and red. Left and right panels respectively show the samples of the 15°C and 23°C common garden experiments. In both experiments, we did not detect any outliers.

Fig. S2: Log_2_FC of gene expression between the ancestral and the hot evolved populations at 15°C (blue) and 23°C (red) of the 146 genes involved in the oxidative phosphorylation. Most of these genes are significantly down regulated at 23°C (n=45, ** FDR<0.05 , * FDR<0.1). Even though most comparison are not statistically significant, most of the down regulated genes at 23°C are up-regulated at 15°C. We produced independent plot for each gene showing the variability across our five replicates in a Supplementary File.

Fig. S3: The number of genes statistically plastic in the ancestral and the two groups of evolved populations after down-sampling each library to the same number of reads (14,464,603). These distribution result from 100 independent sampling of the complete libraries. Although the total number of plastic genes is smaller after the down-sampling, the differences between groups are similar.

Fig. S4: The direction of the evolved gene expression change is highly correlated at 15°C and 23°C. Because the genes are unlikely to evolve independently, the χ^2^ assumptions are violated. To overcome this limitation, we generated an empirical distribution of χ^2^ statistics by bootstrapping the genes that had a significant change in plasticity during evolution in the hot environment and still found a more significant correlation for the sign of gene expression change at 15°C and 23°C than expected by chance. This empirical distribution of χ^2^ statistics was obtained by bootstrapping 241 genes 10,000 times from the list of 417 genes that evolved a significant change in plasticity (FDR<0.1, here we did not condition on a significant evolution at 15°C or 23°C). For each list of 241 genes, a χ^2^ test of independence was computed between the direction of the evolved expression change at 15°C and 23°C. The statistic obtained using the genes that evolved increased plasticity (observed data, red line) is larger than 99% of the statistics obtained by bootstrapping (the blue line shows the 95% threshold). This test indicates that the negative correlation observed for the list of genes showing increasing plasticity could not be obtained from a random sampling of genes showing overall plasticity evolution. Here we assume that both lists of genes have equal modularity: the evolution of a subset of these genes is due to a similar number of causative events.

Fig. S5: Pairwise log2FC of reaction norm slopes between five evolved replicates (as rows and columns). The bottom half displays the genes with decreased plasticity during evolution and the top half the genes with increased plasticity during evolution (see Fig 1-3C with matching color code). There is a strong correlation of the plasticity evolution across all 5 hot-evolved replicates for genes with increased plasticity (top) but not for genes with decreased plasticity (bottom).

Fig. S6: Identification of libraries with female contamination. We summed the expression of the nine chorion genes (CP15 to 19, CP36, CP38) and three yolk proteins (YP1 to 3). We excluded four outlier libraries (in red) with > 1769 counts per million reads for the 12 indicator genes. The retained libraries had < 111 counts per million reads.

Supplementary files 1 & 2:

Reproducibility of the expression plasticity across replicates. Three enzymes of the glycolysis pathway are shown on Fig. 4C and we provide in Supplementary File 1 and Supplementary File 2 the remaining genes of the glycolysis and oxidative phosphorylation pathways respectively. The ancestral replicate populations are indicated by green dots and the hot evolved populations by red dots. Lines indicate plasticity based on the mean expressions values of the five replicates.
