# Supplementary figures and images for: The Evolution of Phenotypic Plasticity in Response to Temperature Stress
Source: Genome Biol Evol. 2020 Oct 6;12(12):2429–40. doi: 10.1093/gbe/evaa206 (PMC7846148; doi:10.1093/gbe/evaa206)

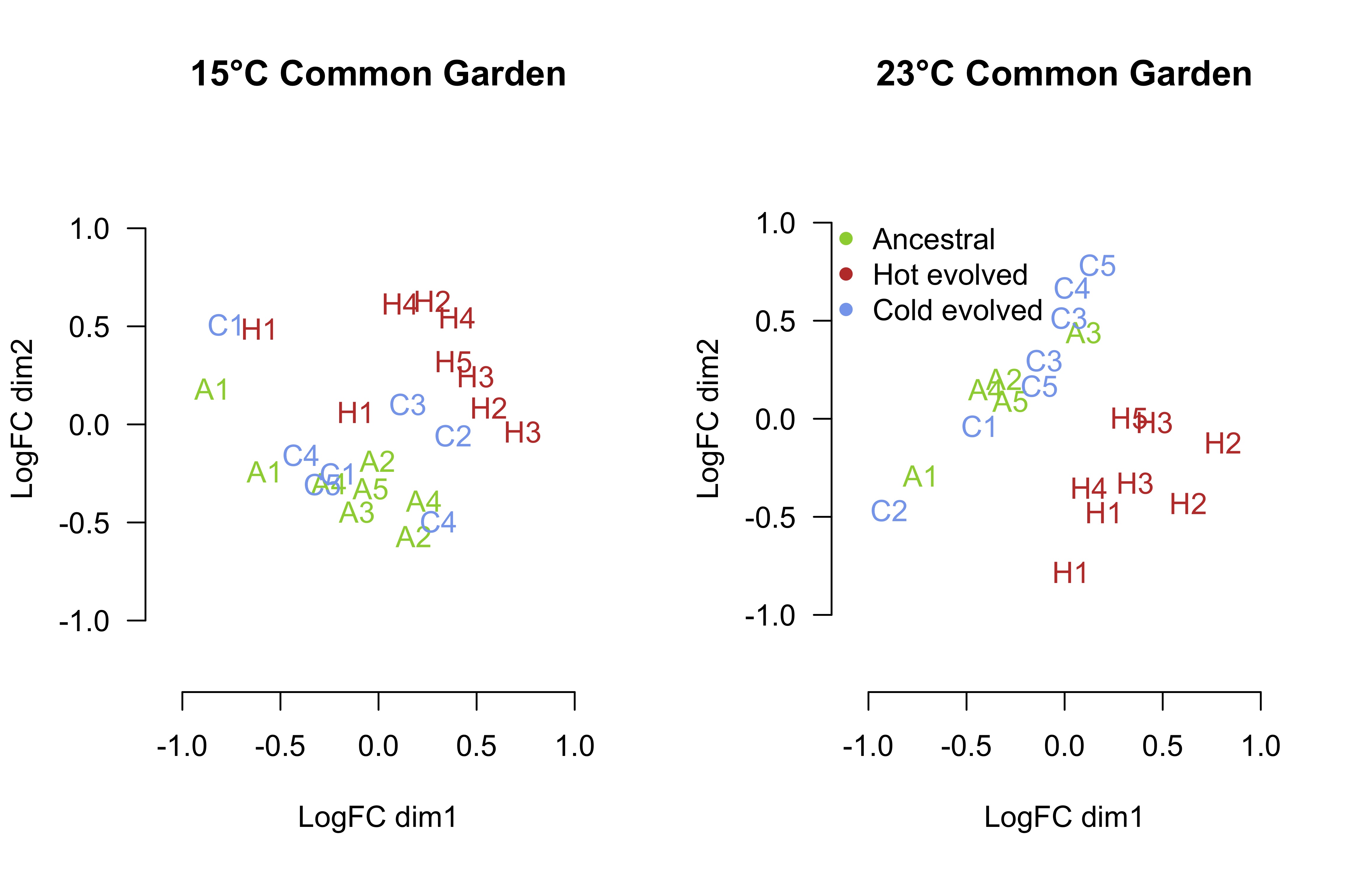

Supplement: evaa206_Supplementary_Data [file evaa206_supplementary_data.zip › FigS1.jpg]

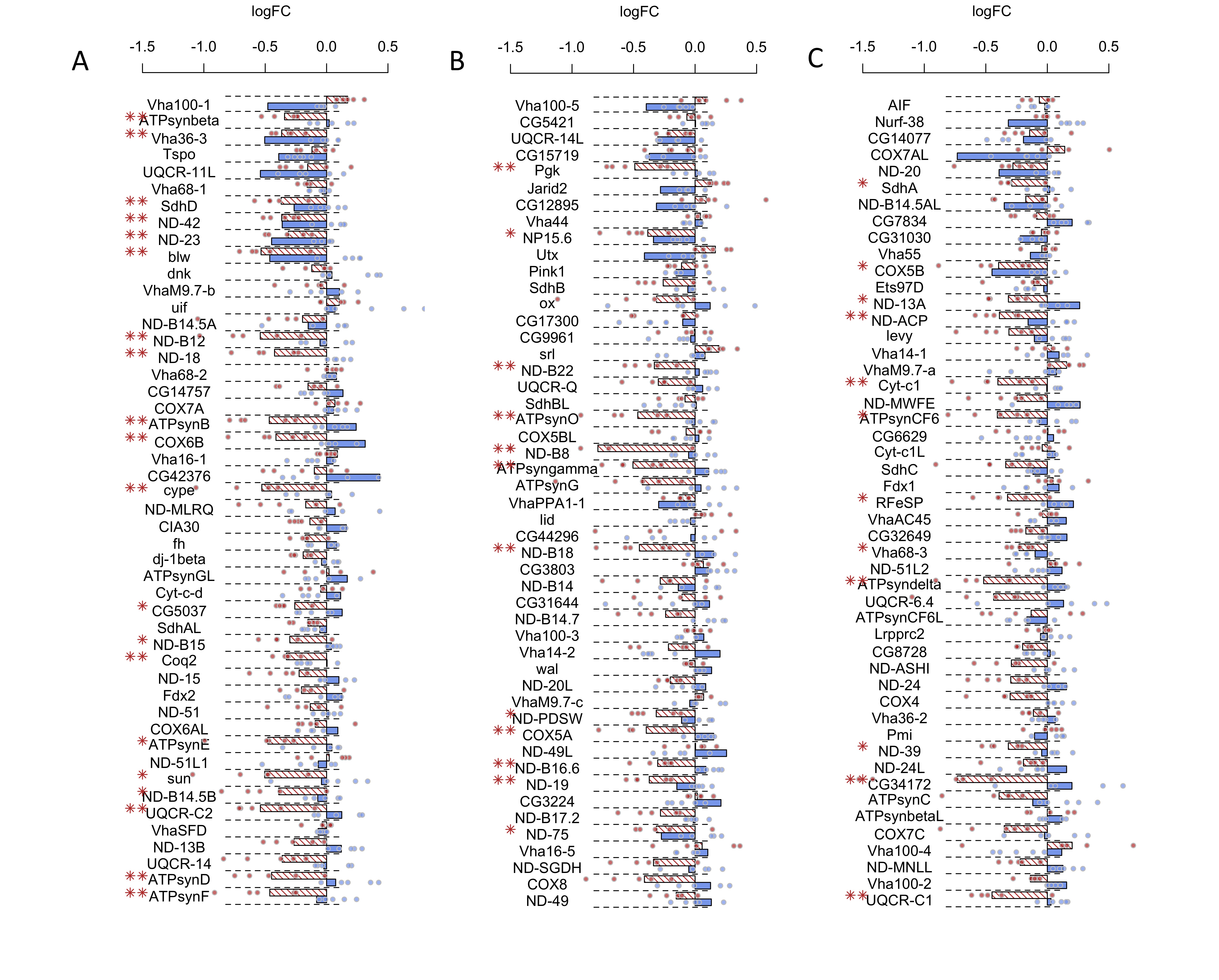

Supplement: evaa206_Supplementary_Data [file evaa206_supplementary_data.zip › FigS2.jpg]

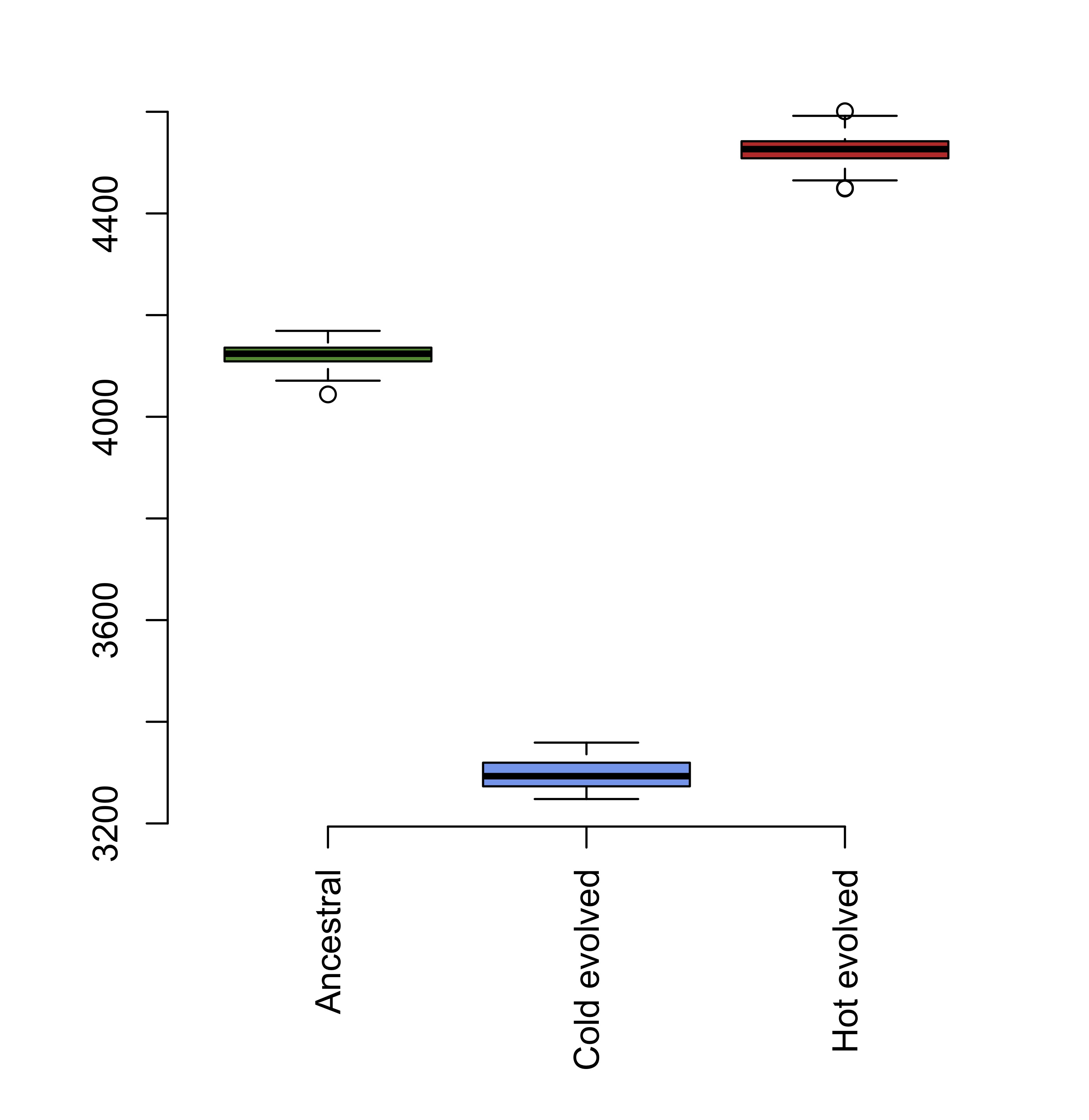

Supplement: evaa206_Supplementary_Data [file evaa206_supplementary_data.zip › FigS3.jpg]

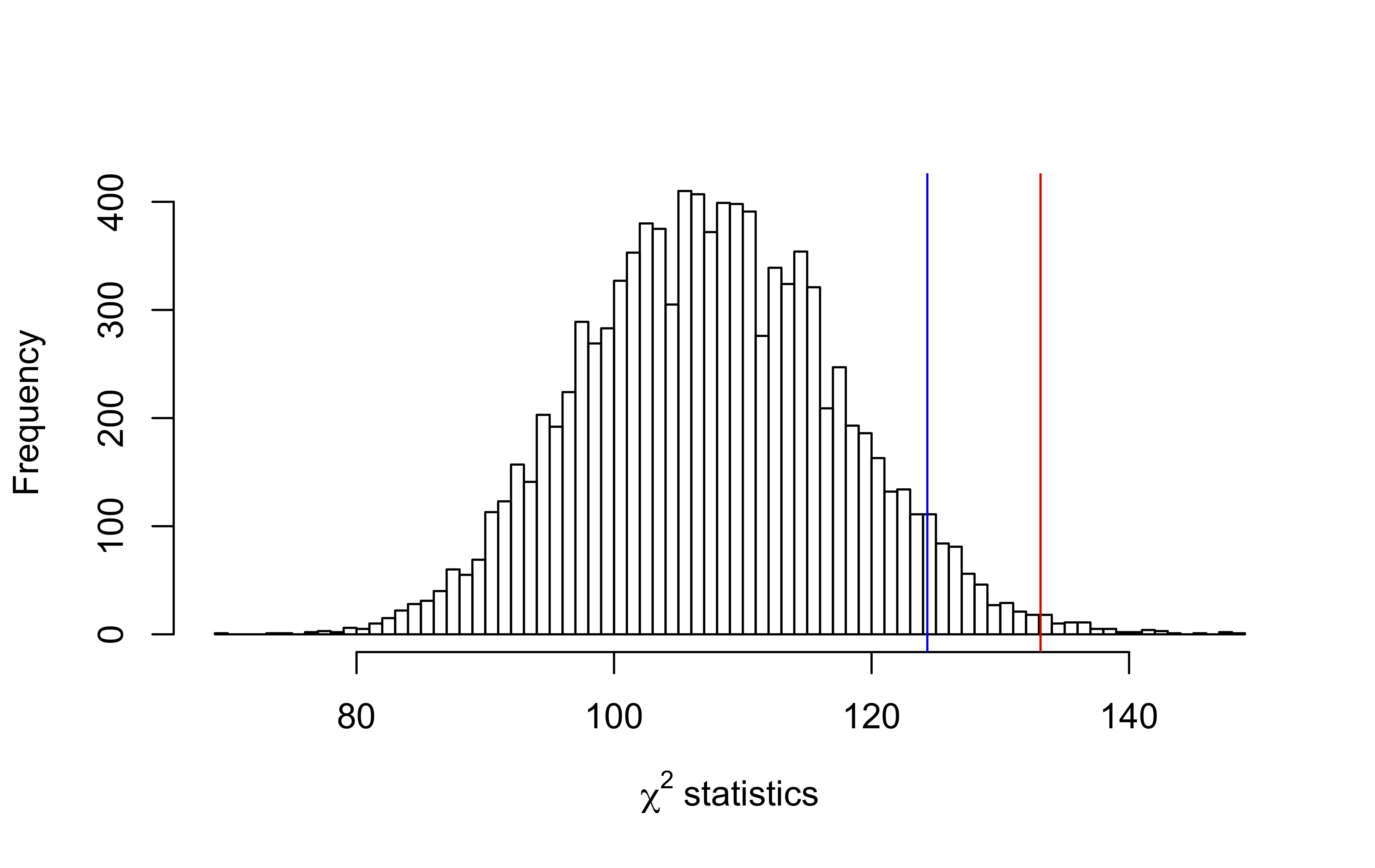

Supplement: evaa206_Supplementary_Data [file evaa206_supplementary_data.zip › FigS4.jpg]

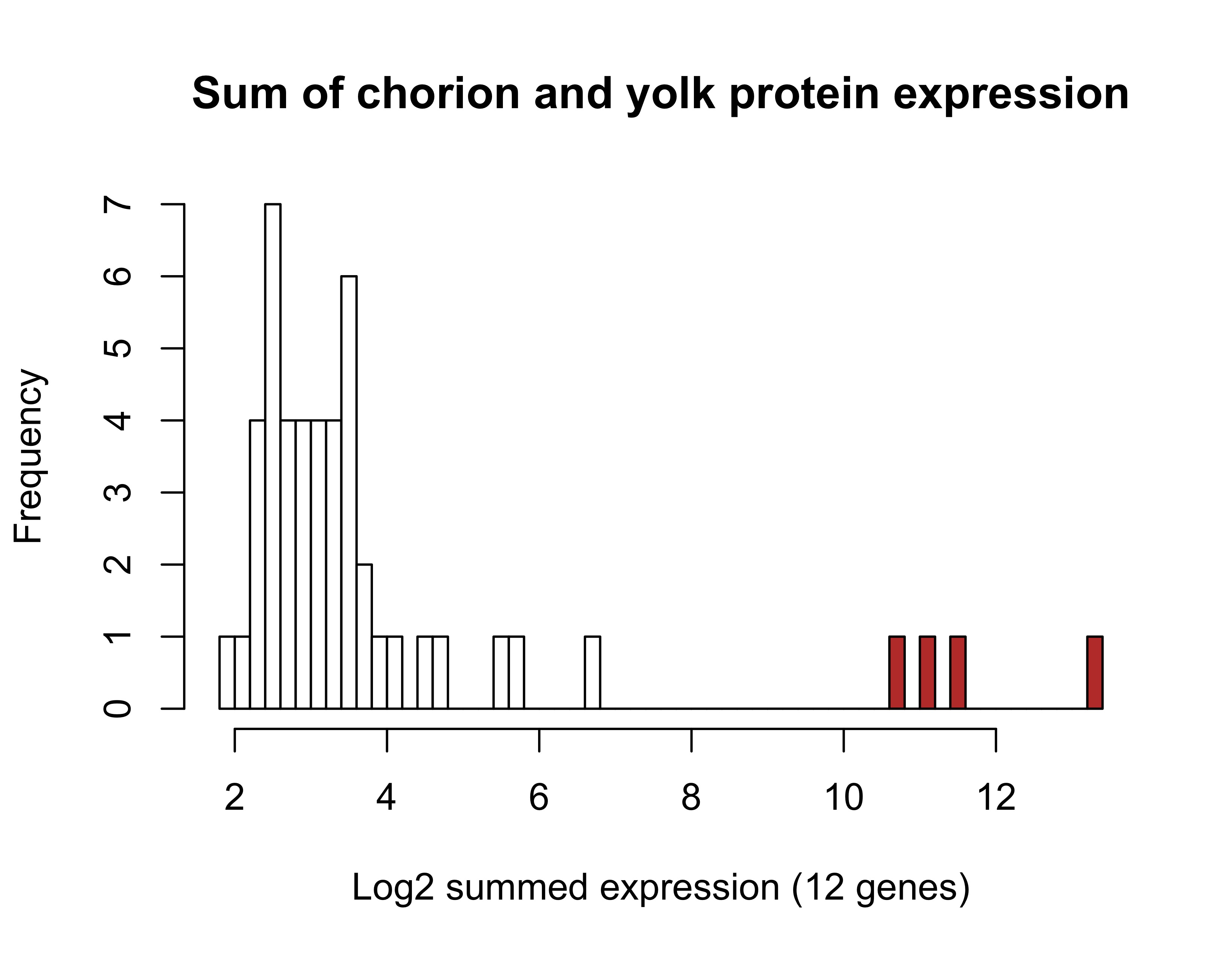

Supplement: evaa206_Supplementary_Data [file evaa206_supplementary_data.zip › FigS6.jpg]

**Hex-A**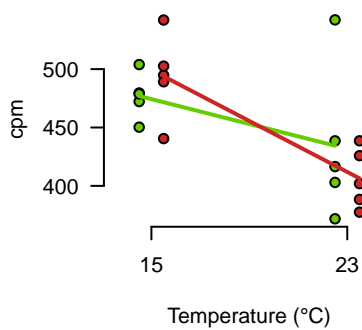**Hex-C**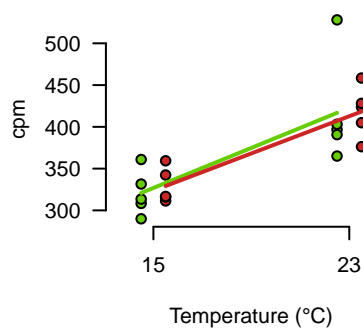**Pgi**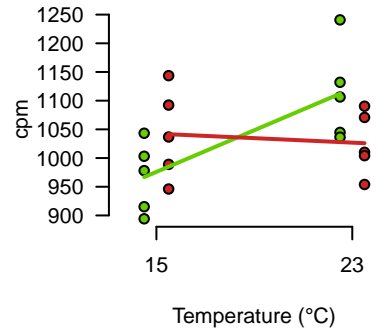**Pfk**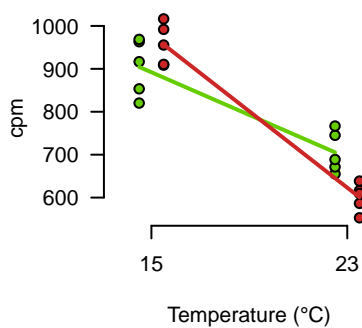**fbp**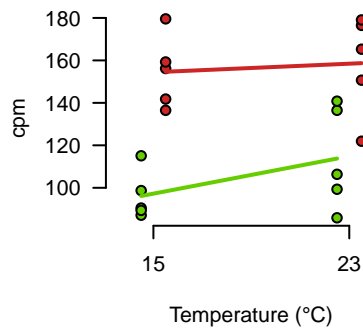**Tpi**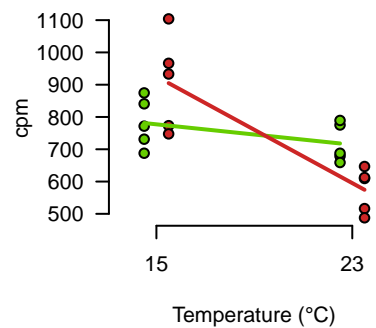**Ald**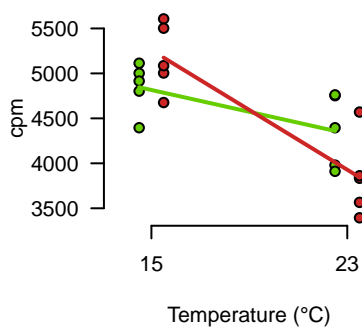**Gapdh1**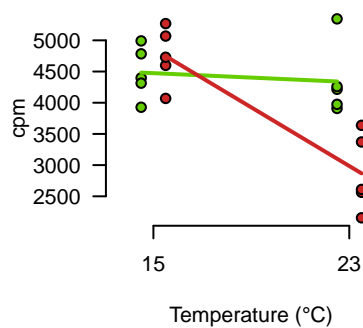**Gapdh2**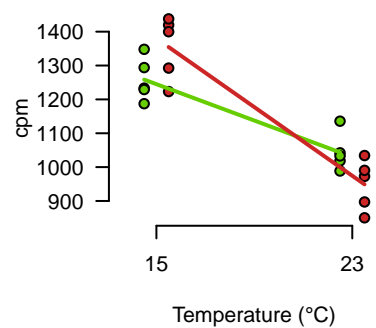

**Pgk**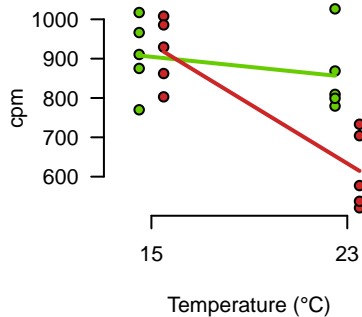**Pglym78**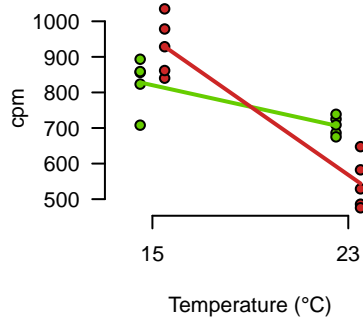**Eno**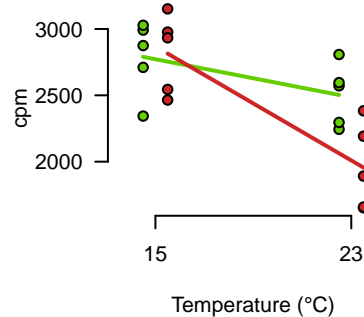**PyK**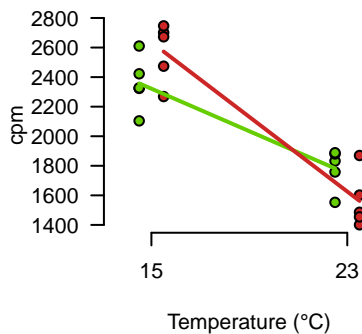**ImpL3**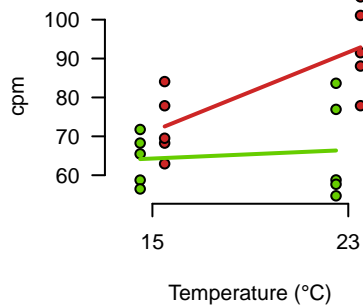**CG11876**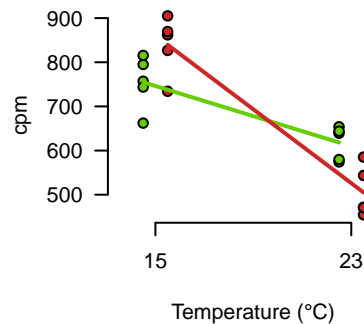**CG5261**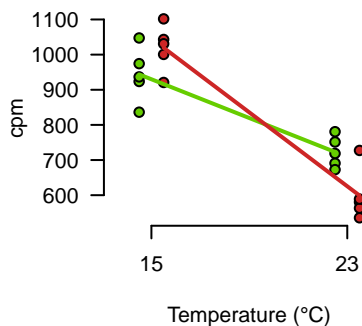**CG7430**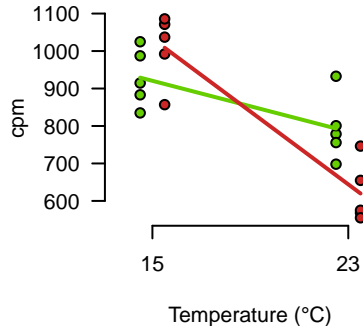**I(1)G0334**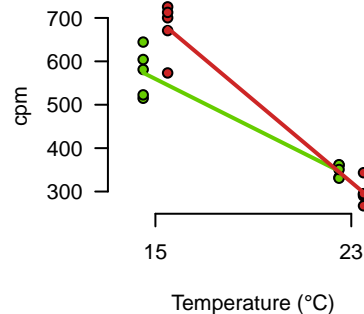

Supplement: evaa206_Supplementary_Data [file evaa206_supplementary_data.zip › Suplementary_File_1.pdf]
